# Loss of sirtuin 1 and mitofusin 2 contributes to enhanced ischemia/reperfusion injury in aged livers

**DOI:** 10.1111/acel.12761

**Published:** 2018-05-17

**Authors:** Sung Kook Chun, Sooyeon Lee, Joseph Flores‐Toro, Rebecca Y. U, Ming‐Jim Yang, Kristina L. Go, Thomas G. Biel, Catherine E. Miney, Schiley Pierre Louis, Brian K. Law, Mary E. Law, Elizabeth M. Thomas, Kevin E. Behrns, Christiaan Leeuwenburgh, Jae‐Sung Kim

**Affiliations:** ^1^ Department of Surgery College of Medicine University of Florida Gainesville FL USA; ^2^ Department of Surgery Saint Louis University St. Louis MO USA; ^3^ Department of Pharmacology & Therapeutics College of Medicine University of Florida Gainesville FL USA; ^4^ Department of Aging and Geriatric Research College of Medicine University of Florida Gainesville FL USA

**Keywords:** aging, autophagy, hepatocytes, liver, mitochondria, mitophagy

## Abstract

Ischemia/reperfusion (I/R) injury is a causative factor contributing to morbidity and mortality during liver resection and transplantation. Livers from elderly patients have a poorer recovery from these surgeries, indicating reduced reparative capacity with aging. Mechanisms underlying this age‐mediated hypersensitivity to I/R injury remain poorly understood. Here, we investigated how sirtuin 1 (SIRT1) and mitofusin 2 (MFN2) are affected by I/R in aged livers. Young (3 months) and old (23–26 months) male C57/BL6 mice were subjected to hepatic I/R in vivo. Primary hepatocytes isolated from each age group were also exposed to simulated in vitro I/R. Biochemical, genetic, and imaging analyses were performed to assess cell death, autophagy flux, mitophagy, and mitochondrial function. Compared to young mice, old livers showed accelerated liver injury following mild I/R. Reperfusion of old hepatocytes also showed necrosis, accompanied with defective autophagy, onset of the mitochondrial permeability transition, and mitochondrial dysfunction. Biochemical analysis indicated a near‐complete loss of both SIRT1 and MFN2 after I/R in old hepatocytes, which did not occur in young cells. Overexpression of either SIRT1 or MFN2 alone in old hepatocytes failed to mitigate I/R injury, while co‐overexpression of both proteins promoted autophagy and prevented mitochondrial dysfunction and cell death after reperfusion. Genetic approaches with deletion and point mutants revealed that SIRT1 deacetylated K655 and K662 residues in the C‐terminus of MFN2, leading to autophagy activation. The SIRT1‐MFN2 axis is pivotal during I/R recovery and may be a novel therapeutic target to reduce I/R injury in aged livers.

## 1 INTRODUCTION

Ischemia/reperfusion (I/R) injury is a fundamental obstacle in liver resection and transplantation surgery. The incidence of liver fibrosis and cancer increases with aging and resection surgery remains as a main treatment option for these patients. With the increasing prevalence of chronic liver disease and metabolic disorder worldwide, surgical intervention is on a rise. Elderly patients are more prone to I/R injury during surgery, presumably due to declined reparative capacities with advancing age. Currently, cellular mechanisms that underlie this age‐dependent I/R hypersensitivity remain poorly understood, and no effective therapies exist to mitigate I/R injury in aged livers.

Our studies have shown that unlike young hepatocytes that readily overcome a mild ischemic insult, the same stress to aged livers is substantially more insidious (Wang et al., 2011). Ischemia/reperfusion hypersensitivity with aging likely involves multiple factors that converge on provoking the onset of mitochondrial permeability transition (MPT), an irreversible mitochondrial damage accompanying cell death. Autophagy is an evolutionally conserved catabolic pathway that clears damaged or surplus cytosolic constituents or dysfunctional mitochondria (Mizushima, Yoshimori & Levine, 2010). Cells with defective autophagy therefore are unable to sequester dysfunctional mitochondria. Accumulation of abnormal mitochondria can damage neighboring normal mitochondria by propagating injurious signals and inducing widespread MPT onset and mitochondrial malfunction (Kim et al., 2008). One key factor behind enhanced I/R injury in aged livers is stimulation of calpains, which leads to the loss of autophagy proteins and subsequent impairment of autophagy (Kim et al., 2013).

Given the central role of autophagy in supporting hepatocellular viability after I/R, we have previously demonstrated in young hepatocytes and livers that an NAD^+^‐dependent deacetylase SIRT1 promotes autophagy and hepatocyte survival after reperfusion (Biel et al., 2016). SIRT1 can activate autophagy through various pathways, including modulation of forkhead box transcription factors (FOX), ATG5, ATG7, LC3, and BECN1 (Huang et al., 2015; Lee et al., 2008; Sun et al., 2015). We recently reported that a gradual depletion of this deacetylase with extended ischemia is a causal to mitochondrial dysfunction and cell death (Biel et al., 2016). Furthermore, SIRT1 overexpression reversed pathological events through its interaction with and subsequent deacetylation of MFN2. This mitochondrial outer membrane protein has diverse functions such as mitochondrial fusion (Chen et al., 2003), tethering between mitochondria and endoplasmic reticulum (ER) (de Brito & Scorrano, 2008; Naon et al., 2016), metabolic regulation (Bach et al., 2003; Sebastián et al., 2012), and the endocytic/secretory pathway (Daniele et al., 2014; Zhao et al., 2012). It is currently unknown how SIRT1 and MFN2 are affected by I/R in old hepatocytes.

In the present work, we find that accelerated loss of both proteins contributes to defective autophagy and poor hepatocyte survival in aged livers after mild I/R stress. We also provide mechanistic insights into how extramitochondrial SIRT1 deacetylates mitochondrial MFN2.

## 2 RESULTS

### 2.1 Age‐dependent sensitivity of livers and hepatocytes to I/R injury

**Figure 1:**
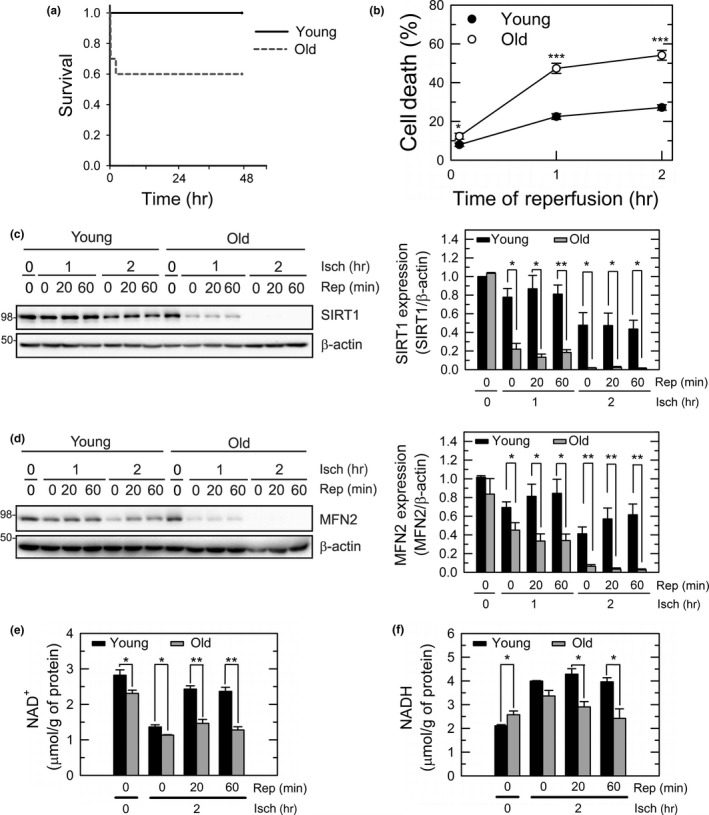
Accelerated loss of SIRT1 and MFN2 after short I/R in old livers. (a) Kaplan–Meier survival analysis of young (*n* = 6) and old mice (*n* = 10), after liver I/R in vivo. The survival probability of old mice after reperfusion was significantly lower than that of young mice (*p* = .025). (b) Cell death in young or old hepatocytes, after 2 hr of ischemia and 5, 60, and 120 min of reperfusion. (c–d) Immunoblotting analysis of SIRT1 and MFN2 expression after ischemia (Isch) and reperfusion (Rep) of hepatocytes. Changes in SIRT1 and MFN2 were normalized to β‐actin. (e–f) Colorimetric analysis of intracellular NAD ^+^ and NADH. I/R, ischemia/reperfusion

As a first step toward understanding effects of aging on I/R injury, we compared survival after hepatic I/R in vivo in 3 months old (referred to as “young” hereafter) or 23–26 months old mice (referred to as “old”). Total liver ischemia was achieved by clamping both suprahepatic inferior vena cava and portal vein for 10 min. Comparison of survival rates after releasing clamp (reperfusion) showed that all young mice were alive after 48 hr, compared to 60% survival in old mice, indicating that old mice are significantly more vulnerable to liver I/R than their younger counterparts (Figures 1a and S1A). As hepatic warm I/R injury prominently occurs to hepatocytes in the pericentral zone of the hepatic lobule (Ikeda et al., 1992), we next isolated primary hepatocytes at both age groups and compared cell death after simulated in vitro I/R (Figure 1b). Cells were exposed to 2 hr of simulated ischemia by exposure to anoxic Krebs‐Ringer HEPES (KRH) solution at pH 6.2. Reperfusion was then induced by reoxygenating anoxic cells in normoxic KRH at pH 7.4 for up to 2 hr (Biel et al., 2016; Kim et al., 2008; Wang et al., 2011). Propidium iodide (PI) fluorometry revealed that greater than 50% cell death occurred in old hepatocytes after 2 hr of reperfusion, while young hepatocytes withstood these conditions quite well with minimal cell death, consistent with our previous report (Wang et al., 2011). Thus, both in vivo and in vitro I/R experiments demonstrate that old livers and hepatocytes are more prone to I/R injury than young counterparts.

### 2.2 SIRT1 is depleted by short ischemia in old hepatocytes

Prolonged I/R depletes young hepatocytes of SIRT1, which in turn causes necrotic cell death after reperfusion (Biel et al., 2016). To examine how short I/R affects SIRT1 in old hepatocytes, we assessed changes in SIRT1 expression before and after ischemia in young and old hepatocytes (Figure 1c). Following 1 or 2 hr of ischemia, cell lysates were collected and immunoblotted after reperfusion. Prior to ischemia, basal levels of hepatic SIRT1 were comparable between the two groups. After 1 hr of ischemia, SIRT1 expression diminished by approximately 80% in reperfused old cells, whereas only 10% decrease was observed in young cells. Although SIRT1 expression further decreased in both ages after 2 hr of I/R, its loss was much more pronounced in old hepatocytes. The expression of SIRT1 was indeed barely detectable after 2 hr of reperfusion in old cells. Measurement of intracellular NAD^+^, an essential factor for SIRT1 activation, showed that levels of NAD^+^ were significantly lower in old cells than young cells before, during and after ischemia (Figure 1e). In addition, while young cells recovered NAD^+^ to near basal levels during reperfusion, old cells did not. Parallel experiments also indicated that NADH levels were significantly lower in old cells after reperfusion (Figure 1f). Thus, SIRT1 activity in reperfused old cells is likely minimal due to the loss of both SIRT1 expression and NAD^+^.

### 2.3 Co‐overexpression of MFN2 and SIRT1 protects old hepatocytes against I/R injury

**Figure 2:**
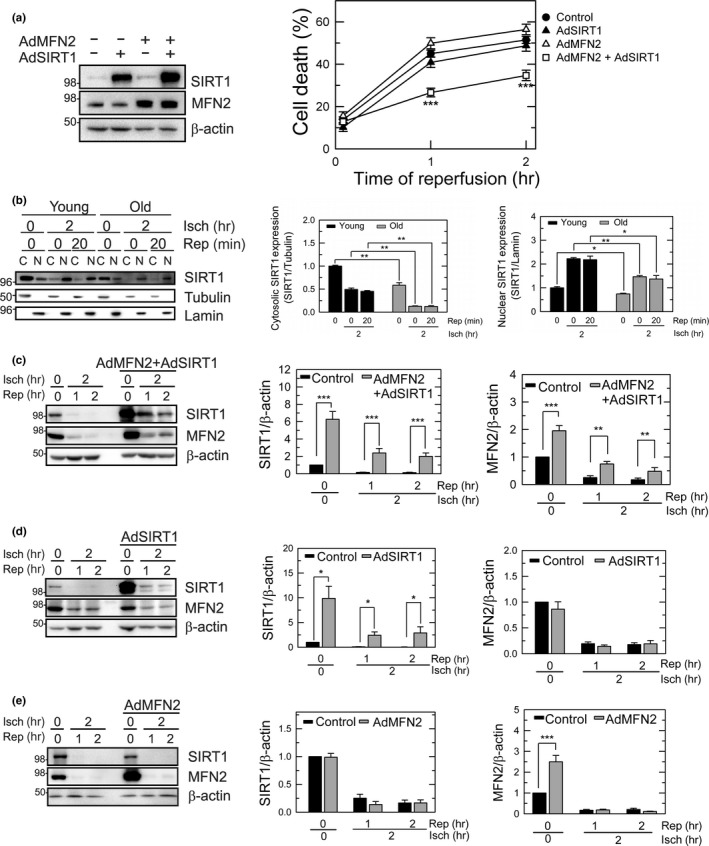
Cytoprotection by co‐overexpression of SIRT1 and MFN2. (a) Cell death after I/R of old hepatocytes. Overexpression of SIRT1 and MFN2 was induced in old hepatocytes by infecting cells with adenoviral SIRT1 (1 MOI) or MFN2 (10 MOI), or in combination. Necrotic cell death was assessed after 2 hr of ischemia. ***p < .001 vs. control. (b) Immunoblotting analysis of changes in cytosolic (C) and nuclear (N) SIRT1 during I/R. Graphs represent quantification of SIRT1 and MFN2 expression relative to subcellular markers. (c–e) Immunoblotting of SIRT1 and MFN2 expression after 2 hr of ischemia and indicated times after reperfusion

To test whether SIRT1 loss is involved in the age‐dependent I/R hypersensitivity, SIRT1 was overexpressed in old hepatocytes by infecting them with adenovirus harboring SIRT1 (AdSIRT1) prior to the onset of ischemia (Figure 2a). Adenovirus expression LacZ was used for a viral control. Cell death assay, however, showed that SIRT1 overexpression alone did not protect old cells against I/R injury, indicating that other factor(s) are attributable for enhanced cell death in reperfused old hepatocytes. We recently reported that SIRT1 interacts with MFN2, a mitochondrial outer membrane protein and that this interaction is required for SIRT1‐dependent cytoprotection against prolonged ischemia (Biel et al., 2016). To examine how aging influences MFN2 expression following short I/R, changes in MFN2 expression were determined in both young and old hepatocytes (Figure 1d). Similar to SIRT1, short I/R significantly and rapidly reduced MFN2 expression in old cells. Approximately 50% reduction in MFN2 from baseline was observed after 1 hr of ischemia. After 2 hr of I/R, MFN2 was further reduced to near‐undetectable levels**.** Overexpression of MFN2 alone with adenoviral delivery (AdMFN2), however, failed to protect old cells after I/R (Figure 2a), implicating that restoration of MFN2 alone is insufficient to suppress the age‐dependent reperfusion injury. Subcellular fractionation revealed that declined expression of cytosolic and nuclear SIRT1 after ischemia was more pronounced in old cells (Figure 2b). To examine whether codepletion of SIRT1 and MFN2 may contribute to I/R hypersensitivity, old cells were treated with both AdSIRT1 and AdMFN2. Co‐overexpression of both proteins significantly diminished cell death after I/R (Figure 2a). Moreover, immunoblotting analysis showed that only co‐overexpression significantly increased the expression of both proteins after I/R (Figure 2c–e). Individual overexpression of SIRT1 or MFN2 alone did not prevent the loss of MFN2 or SIRT1, respectively. Taken together, these results suggest that both SIRT1 and MFN2 are needed for survival after I/R in old hepatocytes.

**Figure 3:**
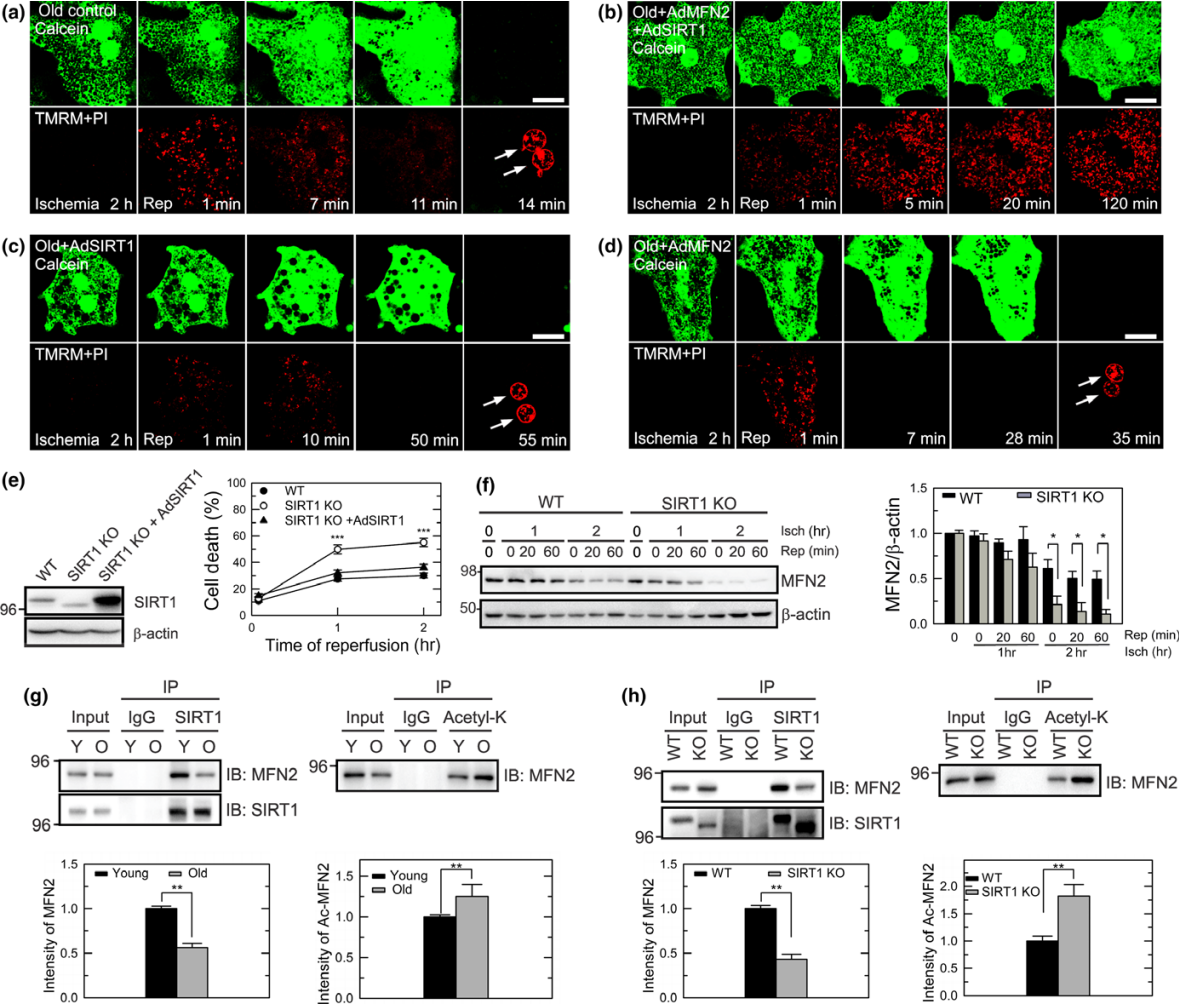
Suppression of the MPT by co‐overexpression of SIRT1 and MFN2 in old hepatocytes. (a–d) Confocal images of the onset of MPT, ΔΨm, and necrosis. Arrows indicate nuclear labeling of PI, indicative of necrotic cell death. Scale bar = 20 μm. (e) Cell death in young WT and liver‐specific SIRT1 KO hepatocytes after 2 hr of ischemia. (f) Immunoblotting analysis of MFN2 expression in young WT and SIRT1 KO hepatocytes after 1 or 2 hr of ischemia. (g) Immunoprecipitation (IP) and immunoblotting (IB) of the mitochondria‐enriched fractions from young and old hepatocytes with SIRT1 (left) or acetylated Lys (right). Graphs represent quantification of SIRT1‐MFN2 immune complexes and acetylated MFN2. (h) IP of SIRT1 and acetylated Lys in young WT and liver‐specific SIRT1 KO hepatocytes

The onset of MPT is a cardinal event culminating in hepatocyte death after I/R (Kim, He, Qian & Lemasters, 2003; Kim, Qian & Lemasters, 2003; Kim et al., 2008). Accordingly, we next employed confocal microscopy to determine how overexpression of SIRT1 or MFN2 alone or in combination affects mitochondria after I/R (Figure 3a–d). MPT onset, mitochondrial membrane potential (ΔΨm), and cell death during I/R were simultaneously visualized using calcein, tetramethylrhodamine methylester (TMRM), and PI, respectively (Kim, He, Qian & Lemasters, 2003 Kim et al., 2008). Calcein is a green fluorescing dye and loads into the cytosol and nucleus but is simultaneously excluded by normal mitochondria whose permeability transition pores remain closed. Tetramethylrhodamine methylester electrophoretically accumulates into mitochondria in response to the negative ΔΨm. As polarized mitochondria take up TMRM while excluding green fluorescing calcein, they appear as small, dark, round voids, each representing a single, functional mitochondrion. After 2 hr of ischemia, TMRM fluorescence was barely detectable due to anoxic depolarization in mitochondria, while mitochondria excluded calcein, indicating a lack of MPT onset during ischemia (Figure 3a). Upon reperfusion, these mitochondria transiently repolarized within 1 min, as judged by TMRM uptake. After 11 min, mitochondria began losing TMRM fluorescence and underwent the MPT, as judged by redistribution of calcein from the cytosol to mitochondria (disappearance of dark voids). In 14 min, cytosolic calcein fluorescence vanished and nuclei became labeled with PI, indicative of cell death (Figure 3a arrows). Consistent with Figure 2a, neither AdSIRT1 nor AdMFN2 alone prevented MPT onset and necrosis (Figure 3c and d). In striking contrast, the MPT and cell death were blocked by co‐overexpression of MFN2 and SIRT1 (Figure 3b). Thus, these data not only indicate that both MFN2 and SIRT1 are indispensable to mitochondrial integrity in reperfused old hepatocytes but also substantiate the importance of mitochondria in the age‐mediated I/R hypersensitivity.

To further test integral roles of SIRT1 in survival of ischemic old hepatocytes, we assessed I/R sensitivity in hepatocytes expressing catalytically inactive SIRT1 with exon 4 deletion. These mice express catalytically inactive SIRT1 (exon 4 deletion) under the control of the albumin promoter; thus, SIRT1 activity is lost exclusively in the liver (Purushotham et al., 2009). In young SIRT1 knockout (KO) hepatocytes at 3 months of age, cell viability after 2 hr of I/R was significantly lower than wild‐type (WT) cells (Figure 3e). Confocal imaging analysis also demonstrated that the onset of MPT and necrosis became evident in reperfused KO cells (Figure S2B), which did not occur to WT cells (Figure S2A). Cell death of these KO hepatocytes was indeed comparable to that of WT old cells (Figure 1b), corroborating the importance of SIRT1 in the age‐dependent I/R injury. Cell viability after overexpression of SIRT1 in these KO cells was restored to similar levels as observed in WT cells. Although basal levels of MFN2 were indiscernible between WT and KO cells (Figure S2C), SIRT1 KO young cells showed a marked loss of MFN2 after 2 hr of I/R (Figure 3f), suggesting accelerated MFN2 loss in SIRT1‐null hepatocytes. However, overexpression of MFN2 alone failed to protect SIRT1 KO cells against I/R injury (Figure S2D).

MFN2 deacetylation by SIRT1 enhances cell viability after I/R (Biel et al., 2016). Although the cellular protection conferred by dual overexpression of MFN2 and SIRT1 supports a functional interaction between these proteins, it is unknown how aging impacts their interaction. To this end, we performed the immunoprecipitation assay using SIRT1 antibody followed by immunoblotting analysis with antibody against MFN2 in mitochondria‐enriched membrane (M) fractions (Figures 3g & S2E). The level of MFN2‐SIRT1 immune complexes considerably decreased in old cells, suggesting reduced MFN2‐SIRT1 interaction with advancing age. Consistently, acetylated form of MFN2 was more prevalent in old cells. To confirm the importance of MFN2‐SIRT1 interaction, immunoprecipitation was repeated in hepatocytes from liver‐specific SIRT1 KO mice (Figure 3h). In young KO cells, levels of MFN2‐SIRT1 complex were significantly reduced, compared to WT cells, while acetylated form of MFN2 was abundant in KO cells. However, the acetylation status of other putative SIRT1 targets such as MFN1, VDAC, FOXO1, FOXO3A, mitoNEET, and PGC1α was not altered by SIRT1 overexpression (Figure S3). Note a visible co‐immunoprecipitation in SIRT1 KO cells due to the presence of a truncated form of SIRT1 in the liver (Purushotham et al., 2009). Collectively, these data suggest that the interaction between SIRT1 and MFN2 declines with aging, leading to enriching acetylated MFN2 in old hepatocytes.

### 2.4 Co‐overexpression of MFN2 and SIRT1 improves autophagic response to I/R in old hepatocytes

**Figure 4:**
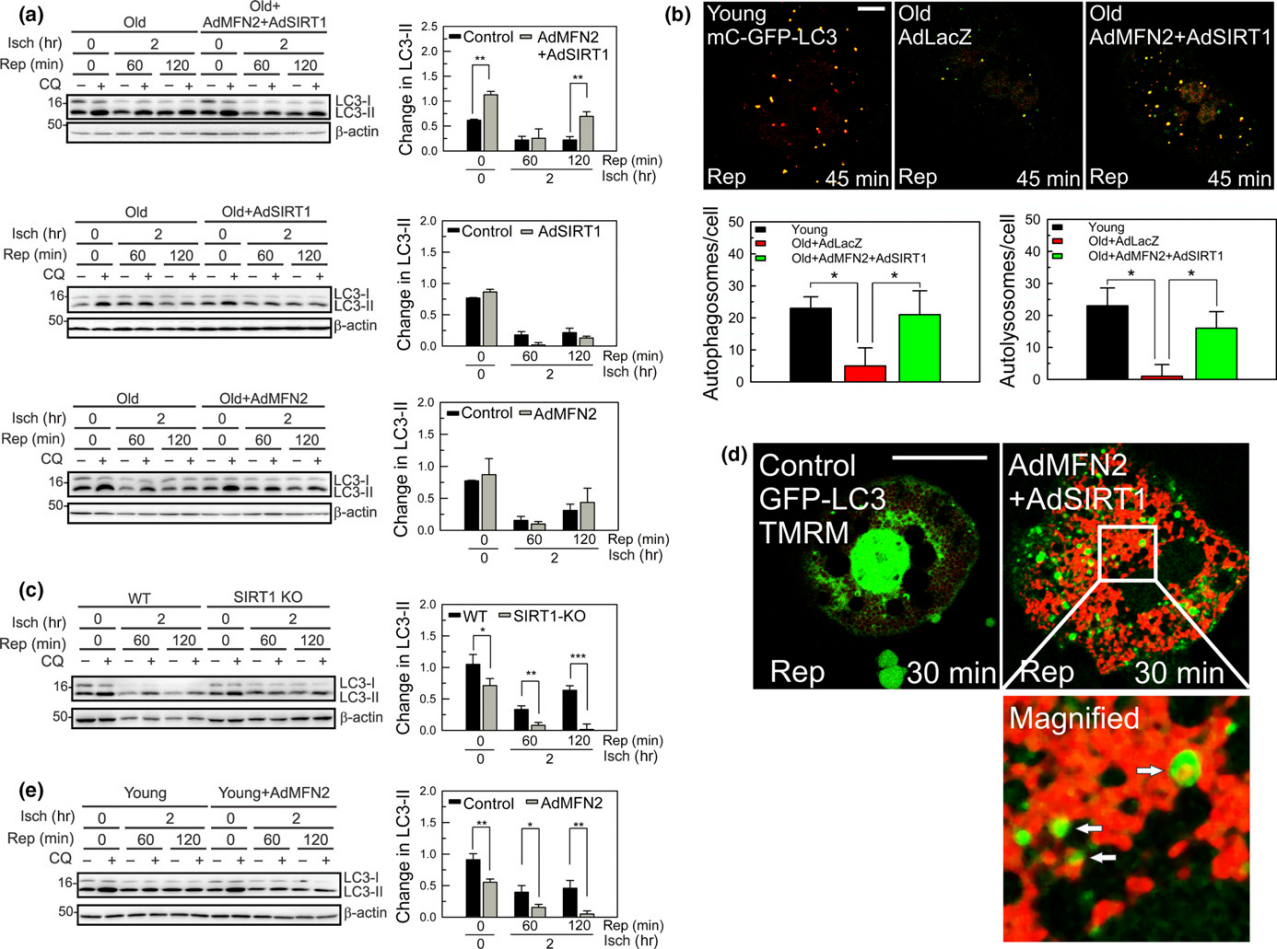
Improved autophagy by dual overexpression of SIRT1 and MFN2. (a) Autophagic flux after 2 hr of ischemia in old hepatocytes. Autophagic flux was measured by immunoblotting LC3‐II levels in the absence or presence of 20 μm chloroquine (CQ). Graph represents quantification of LC3‐II differences before and after the addition of CQ. (b) Representative confocal images of mCherry‐GFP‐LC3 after I/R in young and old hepatocytes. Cells expressing control virus (LacZ) or co‐overexpressing MFN2 and SIRT1 were exposed to 2 hr of ischemia and 45 min of reperfusion. Scale bar = 10 μm. Yellow and red puncta representing autophagosomes and autolysosomes, respectively, were counted after reperfusion. (c) Autophagic flux after I/R in young WT and SIRT1 KO hepatocytes. (d) Representative confocal images of GFP‐LC3 and TMRM after 2 hr of ischemia followed by 30 min of reperfusion. The bottom panel is an enlarged image of the square inset. Arrows indicate the onset of mitophagy. Scale bar = 20 μm. (e) Autophagic flux in ischemic young hepatocytes after MFN2 overexpression

Autophagy activation is an important step for I/R survival, as defective autophagy and consequent failure to sequester damaged mitochondria cause the age‐dependent I/R injury (Wang et al., 2011). To determine whether depletion of MFN2 and SIRT1 during I/R impairs autophagic flux, we overexpressed SIRT1 or MFN2 alone or in combination, and analyzed autophagic flux by comparing the intensity of microtubule associated protein light chain 3‐II (LC3‐II) before and after the addition of chloroquine (CQ) to block degradation by lysosomes (Mizushima et al., 2010) (Figure 4a). Consistent with our previous report (Wang et al., 2011), CQ minimally increased LC3‐II after 2 hr of reperfusion in old hepatocytes. However, co‐overexpression of MFN2 and SIRT1 significantly enhanced autophagic flux before and after I/R. The requirement of both proteins for autophagy stimulation was further supported by our observations where neither SIRT1 nor MFN2 overexpression stimulated autophagy under both basal and I/R condition. We also compared autophagic flux in young and old cells by confocal imaging with the tandem mCherry‐GFP‐LC3 adenovirus (Figure 4b). This dual fluorescent probe provides distinction between autophagosomes (yellow puncta) and autolysosomes (red puncta) as GFP fluorescence becomes quenched in the lumen of autolysosomes due to its acidic pH (Kimura, Noda & Yoshimori, 2007). Concordant with immunoblotting analysis, co‐overexpression of MFN2 and SIRT1 substantially increased the number of both autophagosomes and autolysosomes during reperfusion. Furthermore, SIRT1‐deficient young hepatocytes exhibited subdued autophagic flux after I/R (Figure 4c). In contrast to SIRT1, overexpression of MFN2 in young cells substantially dampened autophagy at baseline and after I/R (Figure 4e), implying that excessive MFN2 could suppress autophagy.

Deficient or impaired mitophagy induces mitochondrial dysfunction after I/R (Biel et al., 2016). We determined whether co‐overexpression mitigates defective mitophagy after I/R. Confocal imaging analysis of GFP‐LC3 in TMRM–labeled hepatocytes revealed that MFN2 and SIRT1 co‐overexpression substantially increased mitophagy, as judged by GFP‐LC3 puncta surrounding mitochondria (Figure 4d**,** arrows).

### 2.5 Ischemia and calpains contribute to MFN2 and SIRT1 depletion during I/R

**Figure 5:**
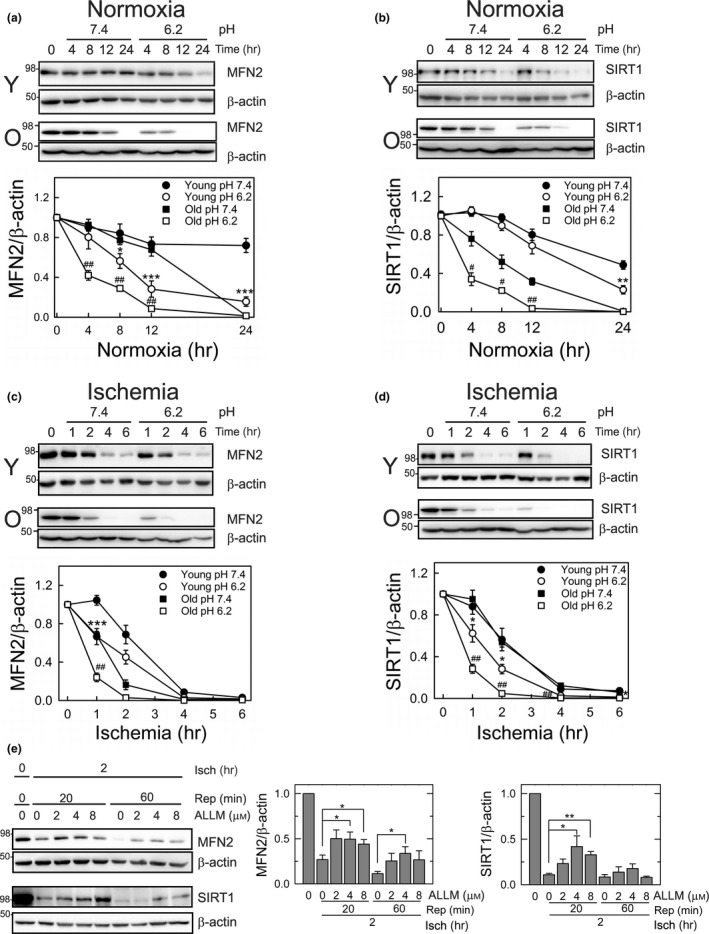
Mechanisms of MFN2 and SIRT1 depletion during I/R. (a–b) pH‐dependent changes in MFN2 and SIRT1 during normoxia in young and old hepatocytes. Immunoblotting analysis in the presence of 35 μm cycloheximide under different pH. *p < .05, **p < .01, and ***p < .001 vs. young cells at pH 7.4. ^#^ *p* < .05 and ^##^ *p* < .01, vs. old cells at pH 7.4. (c–d) pH‐dependent changes in MFN2 and SIRT1 during anoxia in young and old hepatocytes. Effects of pH on MFN2 and SIRT1 expression were determined under anoxic condition with cycloheximide. (e) Loss of MFN2 and SIRT1 by calpains. Changes in MFN2 and SIRT1 expression were assessed with and without calpain inhibitor, ALLM (0–8 μm), after I/R in old hepatocytes. ALLM, *N*‐acetyl‐Leu‐Leu‐methional

We next explored the mechanisms by which MFN2 and SIRT1 are depleted after I/R in old hepatocytes. SIRT1 and MFN2 mRNA levels in old hepatocytes remained unchanged during 2 hr of anoxia or normoxia (Figure S1B and C), indicating that the loss of these proteins occurs independently of transcriptional alterations. To test whether depletion of MFN2 or SIRT1 may be associated with the change in their protein stability, we conducted immunoblotting analysis with the protein synthesis inhibitor, cycloheximide. Under normoxia at pH 7.4, the half‐life time (*t*_1/2_) of MFN2 in young hepatocytes was estimated to exceed 24 hr (Figure 5a). Although old cells had a substantially shorter *t*_1/2_ after prolonged normoxia, MFN2 expression was comparable during the first 12 hr at both ages. We repeated these experiments at pH 6.2 to examine how intracellular acidification, a key event occurring during ischemia, affects both proteins. While MFN2 levels in young cells were stable at neutral pH, lower pH decreased MFN2 levels to approximately 75% of basal values after 12 hr, which was further reduced in old cells. These results indicated that MFN2 in old cells is more vulnerable to acidosis‐mediated destabilization. The half‐life time of SIRT1 in old cells was markedly shorter than that in young cells under normoxia at pH 7.4 (Figure 5b), suggesting that SIRT1 is short‐lived in old hepatocytes. While SIRT1 expression in young cells was similar at both pH, SIRT1 in old cells was significantly destabilized by acidic pH.

To examine how ischemia affects MFN2 and SIRT1, hepatocytes were anoxically incubated at different pH (Figure 5c and d). Under anoxia at pH 7.4, MFN2 became markedly destabilized in old cells even after short ischemia, whereas the changes in SIRT1 were almost identical at both ages, suggesting that MFN2 in old cells is susceptible to anoxic stress. Noticeably, the rate of protein loss was accelerated by acidic environment, particularly during the first 2 hr. After 4 hr, both proteins declined by 98% across neutral or acidic conditions. However, levels of mRNA for SIRT1 and MFN2 were not affected by different pH (Figure S1B and C). Collectively, these results indicate that MFN2 and SIRT1 in old cells are significantly more prone to ischemic stress (anoxia at pH 6.2).

Stimulation of calpains contributes to I/R injury (Biel et al., 2016). To test whether calpain activation is responsible for degradation of MFN2 and SIRT1 in reperfused old cells, old hepatocytes were treated with *N*‐acetyl‐Leu‐Leu‐methional (ALLM), a calpain inhibitor (Figure 5e). *N*‐acetyl‐Leu‐Leu‐methional significantly suppressed both MFN2 and SIRT1 depletion during the early phase of reperfusion. At 1 hr of reperfusion, calpain inhibition delayed MFN2 loss. Altogether, these results suggest that reduced protein stability as well as calpain activation deplete old hepatocytes of MFN2 and SIRT1 during I/R.

### 2.6 Lysine residues in the C‐terminus of MFN2 are targets of SIRT1

**Figure 6:**
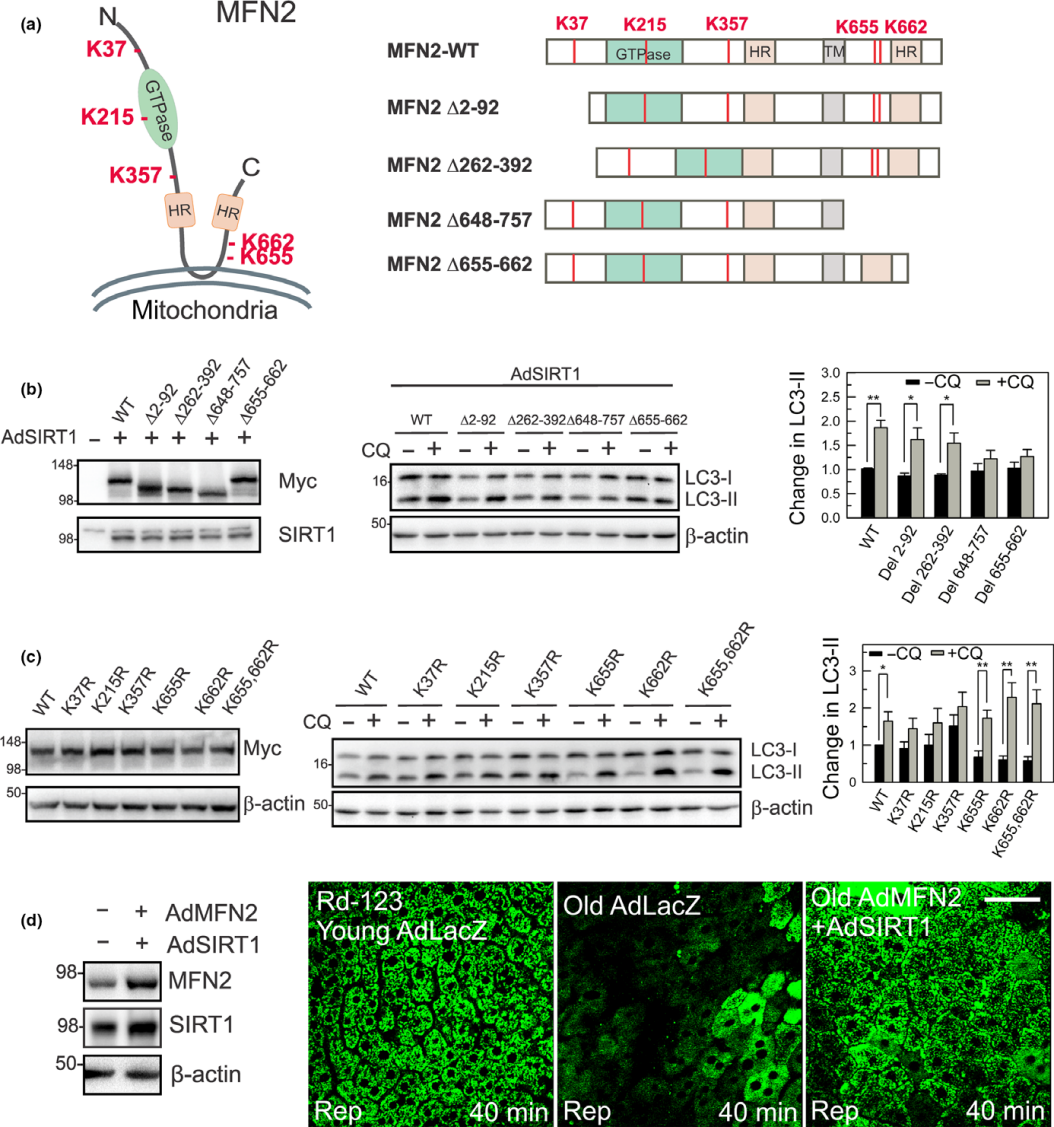
Mechanisms of SIRT1/MFN2‐mediated autophagy. (a) Schematic of MFN2 and putative SIRT1‐targeted Lys resides (K37, K215, K357, K655, K662) with cytosolic heptad repeats (HR). Diagrams of MFN2 deletion constructs. (b) Autophagic flux in MFN2 deletion mutants with SIRT1 overexpression in HEK293T cells. (c) Autophagic flux in MFN2 point mutants without SIRT1 overexpression in HEK293T cells. (d) Multiphoton images of young and old livers after I/R in vivo. After infecting with AdMFN2 and AdSIRT1, old livers were subjected to I/R in vivo. Multiphoton images of Rd‐123 were collected after 40 min of reperfusion. Scale bar = 50 μm

The murine MFN2 contains five putative lysine (Lys) residues (K37, K215, K357, K655, and K662) that meet the criterion as deacetylation sites of SIRT1 (Figure 6a) (Hubbard et al., 2013). To test the relative importance of these Lys residues in their interaction with SIRT1, deletion and point mutants of MFN2 were constructed and expressed in HEK293T cells. As K215 residue lies within the GTPase domain that governs MFN2 functionality (Chen, Chomyn & Chan, 2005; Chen et al., 2003), we generated mutants across the other four Lys residues. The expression of WT or deletion variants of MFN2 was verified by immunoblotting (Figure 6b). To assess which putative target sites of MFN2 are important in SIRT1‐mediated autophagy enhancement, autophagic flux was measured after SIRT1 overexpression. Nicotinamide and trichostatin A were included in order to block deacetylation reactions prior to SIRT1 overexpression (Sun et al., 2015). Deletion mutants in the C‐terminus (Δ648–757 and Δ655–662) significantly blunted SIRT1‐dependent autophagy induction, while N‐terminal mutations (Δ2–92 and Δ262–392) did not (Figure 6b), indicating integral roles of the C‐terminus of MFN2 in SIRT1‐mediated induction of autophagy. To specify C‐terminal Lys residues that are functionally tied to autophagy enhancement, point mutants were generated to mimic Lys deacetylation by substituting Lys with arginine (Arg), which conserves the net positive charge of Lys but prevents charge neutralization by acetylation (Megee, Morgan, Mittman & Smith, 1990). As the C‐terminal sequence between 648 and 757 is likely involved in SIRT1‐dependent autophagy, we reasoned that K‐to‐R substitution in the C‐terminus would enhance autophagy even without SIRT1 overexpression. We generated a single (K655R and K662R) as well as double point mutant (K655, 662R). As shown in Figure 6c, robust autophagy was observed in the K655R, K662R, and K655, 662R mutants even without SIRT1 overexpression. Point mutations in the N‐terminus did not increase autophagy, confirming the results obtained in deletion mutants. Therefore, the acetylation status of C‐terminal Lys residues, particularly K655 and/or K662, may play pivotal roles in mediating SIRT1‐induced autophagy.

### 2.7 Co‐overexpression of MFN2 and SIRT1 protects old livers against I/R injury in vivo

To determine whether MFN2‐SIRT1 axis contributes to functional recovery of the liver after I/R in vivo, we overexpressed both proteins in old mice and examined ΔΨm after reperfusion (Figure 6d). Multiphoton imaging with the mitochondrial membrane potential dye rhodamine 123 (Rd‐123) revealed that young livers after I/R had abundant green Rd‐123 puncta, indicative of respiring polarized mitochondria. In stark contrast, reperfused old livers displayed a drastically diminished and diffusive green fluorescence, representing massive mitochondrial depolarization and dysfunction. However, when old mice were treated with both AdMFN2 and AdSIRT1, mitochondrial dysfunction after I/R was substantially mitigated, although some hepatocytes still showed reduced or no Rd‐123 fluorescence. As mitochondrial depolarization is a hallmark of MPT onset, these data suggest that MFN2‐SIRT1 axis is central to mitochondrial functionality in reperfused aged livers.

## 3 DISCUSSION

Ischemia/reperfusion injury is a causative factor of morbidity and mortality during liver resection, transplantation, and hemorrhagic shock. Aged livers tolerate ischemia poorly and have significantly less reparative capacity following reperfusion. Here, we examined the mechanism by which old livers become susceptible to I/R injury. Upon exposure to mild I/R, a condition that young livers tolerate well, old livers quickly underwent mitochondrial dysfunction and hepatocyte necrosis, preceded by the loss of both deacetylase SIRT1 and its mitochondrial outer membrane target MFN2. This rapid codepletion underlies defective or insufficient autophagy and consequent mitochondrial dysfunction during reperfusion. Our conclusion is supported by the following observations: (i) mild (or short) I/R depleted old hepatocytes of both SIRT1 and MFN2, which did not occur in young cells (Figure 1). (ii) Co‐overexpression of both proteins, but not overexpression of the individual protein alone, suppressed age‐mediated autophagy impairment, MPT onset, mitochondrial depolarization, and cell death after reperfusion (Figures 2, 3, 4). (iii) Ablation of liver‐specific SIRT1 sensitized young cells to I/R injury—an event resembling old cells (Figures 3, 4 & S2), and (iv) overexpression of both MFN2 and SIRT2 significantly improved mitochondrial bioenergetics in old livers after I/R in vivo (Figure 6). We also propose cellular mechanisms underlying MFN2 and SIRT1 loss after I/R (Figure 5) and provide new molecular insights into how these two proteins interact (Figure 6). Overall, our results suggest that both SIRT1 and MFN2 are required to support old hepatocyte survivability after I/R.

The importance of SIRT1 in hepatic autophagy regulation has been reported in ischemic livers (Biel et al., 2016), hepatocellular cancer (Xiong et al., 2017), and hepatosteatosis (Song et al., 2015). MFN2 plays a central role in autophagy induction in the liver (Biel et al., 2016), skeletal and cardiac muscle (Chen & Dorn, 2013; Sebastián et al., 2012). Our findings here not only provide mechanistic correlation of SIRT1‐MFN2 axis in autophagy regulation in the liver but also explain why aged livers are intrinsically vulnerable to ischemic stress. While the involvement of SIRT1 in tissue aging has been well documented in various tissues, Zorzano's group recently reported that defective mitophagy and mitochondrial malfunction in skeletal muscles is causatively linked to a progressive loss of MFN2 with aging (Sebastián et al., 2016), substantiating an integral role of MFN2 in aging and mitophagy. Unlike the muscle, the expression of basal MFN2 or SIRT1 in old hepatocytes was comparable to that in young counterparts. However, we noticed that basal MFN2 in old cells existed in more acetylated forms than young cells, although little cell death occurred at both ages under normoxia (data not shown). The increased acetylated MFN2 in old cells might result from its reduced interaction with SIRT1 and/or decreased SIRT1 activity due to lower NAD^+^ levels. While the acetylation status of MFN2 might minimally affect cell viability under basal conditions, deacetylated MFN2 appears indispensable under stress conditions. The importance of SIRT1‐MFN2 axis in stress response is also supported by our finding that SIRT1 KO hepatocytes undergo necrosis after I/R, while they tolerate normoxia well. Excess MFN2 alone in the absence of SIRT1 was unable to recover cell viability. Indeed, it was as detrimental as insufficient MFN2 in autophagy onset. Numerous mitochondrial proteins are subject to reversible Lys acetylation, many of which are directly involved in metabolic homeostasis (Anderson & Hirschey, 2012; Kim et al., 2006). Thus, it is not surprising to observe that hyperacetylation of mitochondrial proteins is often observed in obesity (Hirschey et al., 2011) and chronic alcohol consumption (Shepard, Tuma & Tuma, 2010). Our results further support that mitochondrial protein modification by the acetylation/deacetylation reaction is a key event governing cellular response to ischemic stress.

MFN2, a mammalian homologue of drosophila FZO, was initially identified as a pro‐fusion factor in mitochondria (Hales & Fuller, 1997). Growing evidence indicates that this mitochondrial shaping protein has additional functions, including regulation of mitochondrial bioenergetics, autophagy, cell cycle, and unfolded protein response (Schrepfer & Scorrano, 2016). Although MFN2 has been shown to modulate autophagy, our findings underscore the importance of MFN2 acetylation status in the onset of hepatocellular autophagy. One potential mechanism behind MFN2‐induced autophagy could be linked to its unique localization at the mitochondria‐ER contact site, which is considered as a source for autophagosomal membranes during elongation (Hailey et al., 2010). Absence of mitochondria‐ER interface proteins such as MFN2 and phosphofurin acidic cluster sorting protein 2 (PACS2) prevents proper autophagosome formation in response to autophagic stimuli including starvation (Hamasaki et al., 2013) or ER stress (Muñoz et al., 2013). Moreover, in a recent study, the ER acetylation machinery directly regulates autophagy in the brain (Peng et al., 2016). Interestingly, PACS2 interacts with SIRT1 and undergoes SIRT1‐mediated deacetylation (Atkins et al., 2014). Therefore, the severe reduction of MFN2 expression during ischemia can alter the integrity of mitochondria‐ER interaction, thereby disrupting the platform for autophagosome or mitophagosome formation, although some reports do not support this tethering function of MFN2 (Cosson, Marchetti, Ravazzola & Orci, 2012; Filadi et al., 2015, 2017). Alternatively, pathologically low levels of MFN2 may adversely affect the delivery of autophagosomes to lysosomes, as MFN2 may be involved in the fusion between the autophagosome and the lysosome through its interaction with the Ras‐related protein Rab7 (Zhao et al., 2012). On the other hand, as MFN2 ubiquitination by PTEN‐induced kinase 1(PINK1) serves as a mitophagy signal for identifying and clearing damaged mitochondria (Chen & Dorn, 2013), depletion of MFN2 during I/R could prevent PINK1‐associated mitophagy in old livers. Noticeably, I/R also significantly decreased MFN1 expression in old hepatocytes (Figure S4A). However, MFN1 overexpression aggravated cell killing in reperfused old hepatocytes (Figure S4B). Moreover, SIRT1 did not alter the acetylation status of this isoform (Figure S3) (Biel et al., 2016). Autophagic flux and imaging analysis also revealed that co‐overexpression of MFN1 and SIRT1 did not influence autophagy and onset of MPT in both ages (Figure S5), suggesting a minimal role of MFN1 in I/R injury to old cells.

What accounts for MFN2 loss during I/R? Our data suggest that calpain activation and cellular acidosis may be important factors. Detrimental effects of calpain activation on hepatocyte viability after I/R have been shown in both young and old livers (Kim et al., 2013; Wang et al., 2011). Calpain activation degrades MFN2 in neurons (Wang et al., 2015). Despite its involvement in MFN2 loss, blockade of calpains was not sufficient to restore MFN2 to basal levels, indicating the presence of additional factor(s). Our results implicate that tissue acidosis may be another player. Anoxia and acidosis are cardinal features of hepatic ischemia. Alterations in pH can perturb activity, structure, and conformation of proteins (Honig & Nicholls, 1995). As mitochondria have an alkaline environment, one possibility is that acidic pH could destabilize mitochondrial proteins. However, this may not be the case as other mitochondrial outer membrane proteins such as mitochondrial Asn‐Glu‐Glu‐Thr (MitoNEET) and voltage‐dependent anion channel (VDAC) were not affected by acidosis (data not shown), implying that MFN2 may specifically be targeted by low pH. MFN2 comprises a bipartite transmembrane region and a highly conserved heptad repeat (HR) domain that contains a helical, coiled‐coil structure near the C‐terminus (Figure 6a). Crystallographic analysis revealed that MFN2 can form either homotypic or heterotypic dimer through HR‐mediated interaction (Koshiba et al., 2004). As changes in pH profoundly impact the hydrogen bonds and electrostatic properties that are essential to the maintenance of stable protein complexes, acidic pH could induce a conformational change, leading to weakening the MFN1‐MFN2 or MFN2‐MFN2 interaction. Enhanced transition of a dimer to monomers by decreasing pH has been observed in dynein protein (Mohan, Barve, Chatterjee & Hosur, 2006). Future studies are warranted to determine why acidic environment promotes MFN2 degradation.

Based on the current knowledge, MFN2 is the first bona fide mitochondrial protein that can be deacetylated by SIRT1. Among the five potential Lys targets of SIRT1, our findings point out that the C‐terminal Lys residues of MFN2 are critical acetylation/deacetylation sites that mediate SIRT1‐dependent autophagy. In our recent report, deletion of either 2–92 or 262–396 sequences of MFN2 blunted autophagy induction by SIRT1, implying that two areas adjacent to the N‐terminus domain are also important in SIRT1‐mediated autophagy induction (Biel et al., 2016). The present data with deletion and point mutation approaches clearly indicate that Lys 655 and 662 are two important deacetylation residues. We observed a substantial reduction in MFN2‐SIRT1 interaction when N‐terminal mutants of MFN2 were co‐immunoprecipitated with SIRT1 (data not shown). Thus, it is plausible that each terminus may have distinct function. We postulate that the C‐terminal domain of MFN2 may act as a substrate for SIRT1, while the N‐terminus may be important in MFN2‐SIRT1 interaction. Functional difference between two termini of MFN2 has previously been proposed (Chen et al., 2014; Rojo, Legros, Chateau & Lombès, 2002).

While our studies demonstrate an integral role of hepatocytes in the early phase of reperfusion injury, it is noteworthy that a sterile inflammation with the activation of innate immune cells through the release of damage‐associated molecular patterns (DAMP) potentiates reperfusion injury in the liver (Jaeschke, 2011). DAMP are known to activate Kupffer cells and recruits activated neutrophils and monocytes into the liver, which promotes not only ROS generation in these phagocytes but also mitochondrial dysfunction in hepatocytes, leading to worsening hepatocyte injury. How SIRT1 and MFN2 affect nonparenchymal cells during I/R will require future study.

In conclusion, mild I/R depletes old hepatocytes of both SIRT1 and MFN2, leading to defective autophagy, MPT onset, mitochondrial dysfunction, and cell death. Co‐overexpression of SIRT1 and MFN2 rescues old hepatocytes after reperfusion through the interaction between two proteins. MFN2‐SIRT1 axis could be a new therapeutic target to ameliorate hepatic function after liver resection or transplantation of the elderly patient.

## 4 EXPERIMENTAL PROCEDURES

### 4.1 Animals

Three‐month and 23‐ to 26‐month‐old male C57/BL6 mice (National Institute of Aging) were fed with standard chow ad libitum. All animal work was performed under the approval of the Institutional Care and Use Committee of the University of Florida. Mice with liver‐specific deletion of functional SIRT1 carry SIRT1 allele floxed exon 4 and express the Cre recombinase driven by the albumin promoter, as previously described (Purushotham et al., 2009).

### 4.2 In vivo I/R

Global hepatic ischemia was achieved by occluding both suprahepatic inferior vena cava and portal vein with a microvascular clamp for 10 min. Reperfusion was initiated by removing the microvascular clamp. For survival analysis, mice were monitored in individual cages for up to 48 hr with analgesics provided as necessary.

### 4.3 Hepatocyte isolation and culture

Primary hepatocytes from male C57BL/6 mice at young and old age were isolated with collagenase perfusion protocol, as previously reported (Biel et al., 2016; Kim, He, Qian & Lemasters, 2003; Kim et al., 2013; Wang et al., 2011). Hepatocytes with viability of greater than 80% were used for every experiment, as determined trypan blue exclusion. Cells were cultured overnight in Waymouth's medium and used the following day for experiments. For cycloheximide experiments, cells were incubated with 35 μm cycloheximide in Krebs‐Ringer HEPES (KRH) buffer (either pH 7.2 or 6.2) under normoxic or anoxic conditions for up to 24 hr or 6 hr, respectively. For calpain inhibition experiments, cells were incubated with 1–10 μm ALLM for 16 hr prior to simulated anoxia, and continuously through I/R.

### 4.4 Simulated in vitro I/R

The combination of anoxia and acidic pH is a key feature of ischemia. To simulate in vitro ischemia, hepatocytes were incubated for 2 hr in KRH buffer at pH 6.2 and 37°C, in an anaerobic chamber (Coy Laboratory Products; Ann Arbor, MI, USA). For simplicity, we henceforth refer to this simulation as in vitro ischemia. Reoxygenation and restoration of physiologic pH during reperfusion were achieved by replacing acidic anoxic KRH buffer with aerobic KRH buffer at pH 7.4 at 37°C (Biel et al., 2016; Kim, He, Qian & Lemasters, 2003; Kim et al., 2013; Wang et al., 2011). Changes in SIRT1, MFN2, and GAPDH mRNA were determined with quantitative real‐time polymerase chain reaction (RT–PCR), as previously described (Biel et al., 2016). The primer sequences of individual genes are shown in Table S1.

### 4.5 Adenoviral overexpression of MFN2 and SIRT1

Adenovirus encoding SIRT1 (AdSIRT1) was kindly provided by Dr. Sadoshima (Rutgers New Jersey Medical School, Newark, NJ, USA). Hepatocytes were infected with AdSIRT1 (1–10 MOI) or AdMFN2 (up to 20 MOI) in a hormonally defined medium (HDM) for 2 hr, after which the medium was replaced with fresh Waymouth's medium and incubated overnight (Biel et al., 2016). For MFN2 and SIRT1 co‐overexpression, hepatocytes were sequentially infected with AdMFN2 (5 to 20 MOI) for 2 hr and AdSIRT1 (1 to 5 MOI) for an additional 2 hr. For imaging analysis of autophagy, AdGFP‐LC3 or AdmCherry‐GFP‐LC3 was used, as previously described (Biel et al., 2016). For all experiments, adenovirus expressing GFP or LacZ was used as a viral control. For adenoviral delivery in vivo, mice were intraperitoneally injected with 10 μl of 10^12^ viral particles of AdSIRT1 and AdMFN2 or AdLacZ.

### 4.6 Cell death and NAD^+^ assay

Necrosis was assessed by a PI fluorometry using a multiwell fluorescence scanner (SpectraMax M2; Molecular device, Sunnyvale, CA, USA), as previously described (Kim, Qian & Lemasters, 2003; Nieminen et al., 1992). Intracellular levels of NAD^+^ and NADH were colorimetrically determined with a commercial kit (AAT Bioquest, Sunnyvale, CA, USA).

### 4.7 Confocal microscopy

Confocal images of TMRM, calcein, PI, GFP‐LC3, and mCherry‐GFP‐LC3 were collected with a gas‐tight chamber (Zeiss, Jena, Germany) using an inverted Zeiss 510 laser scanning confocal microscope, as previously described (Biel et al., 2016).

### 4.8 MFN2 mutant plasmids and cell transfection

QuikChange II mutagenesis kit (Agilent Technologies, Santa Clara, CA, USA) was used to delete amino acids 2–92, 262–392, 648–757, or 655–662, or convert specific Lys residues to Arg in MFN2‐Myc (Addgene plasmid # 23213). The oligonucleotides used for each deletion mutant or point mutation are described in Table 1. For transfection into HEK293T cells, MFN2 plasmids were complexed with PolyJet Transfection Reagent (SignaGen Laboratories, Rockville, MD, USA) and added to cells in serum‐depleted Dulbecco's modified Eagle's medium (DMEM) for 8 hr, then changed to fresh DMEM with 2% FBS containing 25 μm trichostatin A and 5 mm nicotinamide) for 8 hr to maximize protein acetylation (Sun et al., 2015). Some samples were additionally incubated with AdSIRT1 for another 12 hr.

**Table 1:** Primers used to generate MFN2 mutations

Plasmid	Primers
MFN2^Δ2–92^	5′‐CTTACCAGCTAGAAACGAGATGATGAAGGTGGCTTTTTTTGG‐3′ 5′‐CCAAAAAAAGCCACCTTCATCATCTCGTTTCTAGCTGGTAAG‐3′
MFN2^Δ262–392^	5′‐CCTGAACAACCGCTGGGATATGCGGGAAGAGCGGCAAGAC‐3′ 5′‐GTCTTGCCGCTCTTCCCGCATATCCCAGCGGTTGTTCAGG‐3′
MFN2^Δ648–757^	5′‐GTATGGCCTCCTGTACGTCTATGGATCCTCTAGAGGTGAACAAAAGA‐3′ 5′‐TCTTTTGTTCACCTCTAGAGGATCCATAGACGTACAGGAGGCCATAC‐3′
MFN2^Δ655–662^	5′‐GAGCGACTGACCTGGACCACCCGCCAGTTTGTGGAATACGCC‐3′ 5′‐GGCGTATTCCACAAACTGGCGGGTGGTCCAGGTCAGTCGCTC‐3′
MFN2^K37R^	5′‐CACTTTGTCACTGCCAAGAGAAAGATCAATGGAATCTTTG‐3′ 5′‐CAAAGATTCCATTGATCTTTCTCTTGGCAGTGACAAAGTG‐3′
MFN2^L215R^	5′‐GACAGCTGGATTGATAGGTTTTGCCTGGATGCTGATGTG‐3′ 5′‐CACATCAGCATCCAGGCAAAACCTATCAATCCAGCTGTC‐3′
MFN2^K357R^	5′‐GTCTGCAGTAAAGACCAGATTTGAGCAGCACACAGTCCG‐3′ 5′‐CGGACTGTGTGCTGCTCAAATCTGGTCTTTACTGCAGAC‐3′
MFN2^K655R^	5′‐CTGACCTGGACCACCAGAGCCAAAGAGAGGGCCTTCAAG‐3′ 5′‐CTTGAAGGCCCTCTCTTTGGCTCTGGTGGTCCAGGTCAG‐3′
MFN2^K662R^	5′‐CAAAGAGAGGGCCTTCAGGCGCCAGTTTGTGGAATACGC‐3′ 5′‐GCGTATTCCACAAACTGGCGCCTGAAGGCCCTCTCTTTG‐3′
MFN2^K655, 662R^	5′‐CTGACCTGGACCACCAGAGCCAAAGAGAGGGCCTTCAGG‐3′ 5′‐CCTGAAGGCCCTCTCTTTGGCTCTGGTGGTCCAGGTCAG‐3′

### 4.9 Intravital multiphoton microscopy

Livers were labeled with Rd‐123, as previously described (Kim et al., 2008, 2013; Wang et al., 2011). After 20 min of reperfusion in vivo, the liver was gently withdrawn from the abdominal cavity and placed over a glass coverslip on the stage of a Zeiss LSM510 equipped with a multiphoton microscope (Coherent Inc., Santa Clara, CA, USA). Images were collected with a 40× water‐immersion objective lens with excitation of 780 nm. Fifteen to twenty images were randomly collected per liver.

### 4.10 Immunoblotting

Hepatocyte and liver lysates were prepared in radio immunoprecipitation assay (RIPA) buffer supplemented with protease and phosphatase inhibitors. Protein concentrations were determined by Bradford BCA assay, and equal amounts of protein were run on polyacrylamide gels and transferred to either nitrocellulose or polyvinylidene difluoride membranes. Antibodies used are as follows: MFN2 (Abcam #ab56889), MFN1 (Abcam #ab126575), SIRT1 (Millipore #07‐131), LC3B (Cell Signaling, #2775), c‐myc (Santa Cruz Biotechnology 9E10, sc‐40), β‐actin (Santa Cruz Biotechnology #sc‐47778), VDAC (Abcam #ab34726), Calnexin (Santa Cruz Biotechnology #sc‐20), and α‐tubulin (Sigma #T9026). Bands were detected with Lumigen ECL Ultra Chemiluminescent reagents (Lumigen, Southfield, MI) and imaged using a ChemiDoc MP System (Bio‐Rad, Hercules, CA, USA). Densitometry for changes in protein expression was determined using ImageJ software (National Institutes of Health, Bethesda, MD, USA).

### 4.11 Immunoprecipitation

Mitochondria‐enriched membrane fractions were prepared as described (Biel et al., 2016). Acetylated Lys‐conjugated agarose beads (ImmuneChem # ICP0388, British Columbia, Canada) were mixed with protein extracts in RIPA buffer and rotated at 4°C overnight. Beads were centrifuged 1,000×*g*, 5 min, and supernatant was stored separately as unbound control. Beads were rinsed three times by resuspension in 500 μl RIPA buffer, then centrifuged and boiled in sample buffer for 5 min for immunoblotting. SIRT1 immunoprecipitation was carried out, as previously described (Biel et al., 2016).

### 4.12 Data analysis

All experiments are representative of at least three different cell isolations or animals per group. Mean ± standard error (SEM) was calculated and significance determined by performing unpaired two‐tailed Student's *t* tests, using SigmaPlot (version 10). *p*‐Values <.05 (*), <.01 (**) and <.001 (***) were considered significant.

## CONFLICT OF INTEREST

All authors declare no conflict of interest.

## CONTRIBUTIONS

SL, SKC, and J‐SK designed the experiments and performed the analysis and data interpretation. SL, SKC, JF, RYU, TGB, MJY, KLG, CEM, and SPL performed experiments. JF, MEL, and BKL generated MFN2‐mutant plasmids. SL and J‐SK wrote and edited the manuscript. EMT, CL, and KEB discussed data and provided intellectual contributions.
