# Supplementary figures and images for: Loss of sirtuin 1 and mitofusin 2 contributes to enhanced ischemia/reperfusion injury in aged livers
Source: Aging Cell. 2018 May 17;17(4):e12761. doi: 10.1111/acel.12761 (PMC6052398; doi:10.1111/acel.12761)

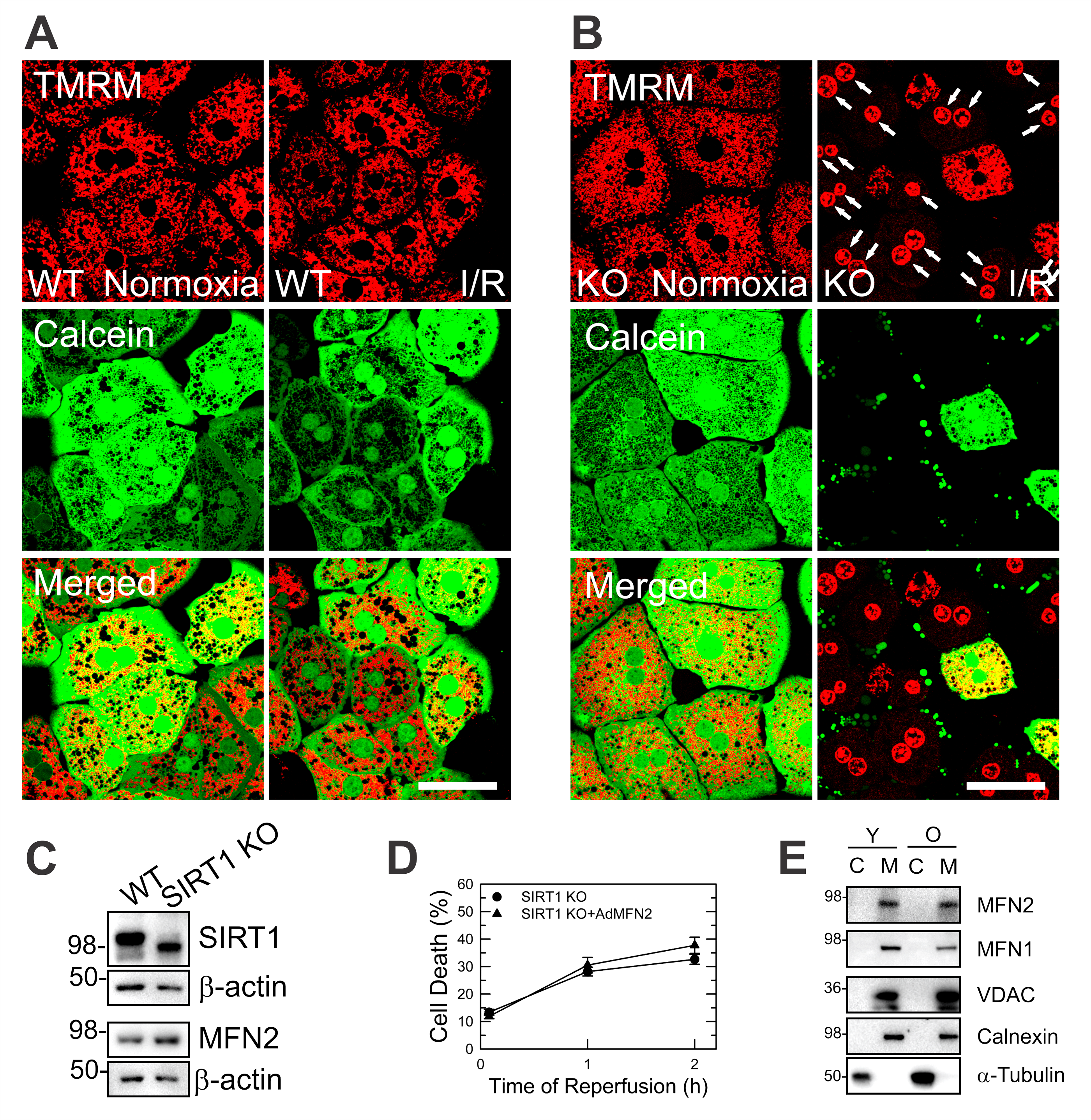

Supplement: Supplementary file 2 [file ACEL-17-na-s002.tif]

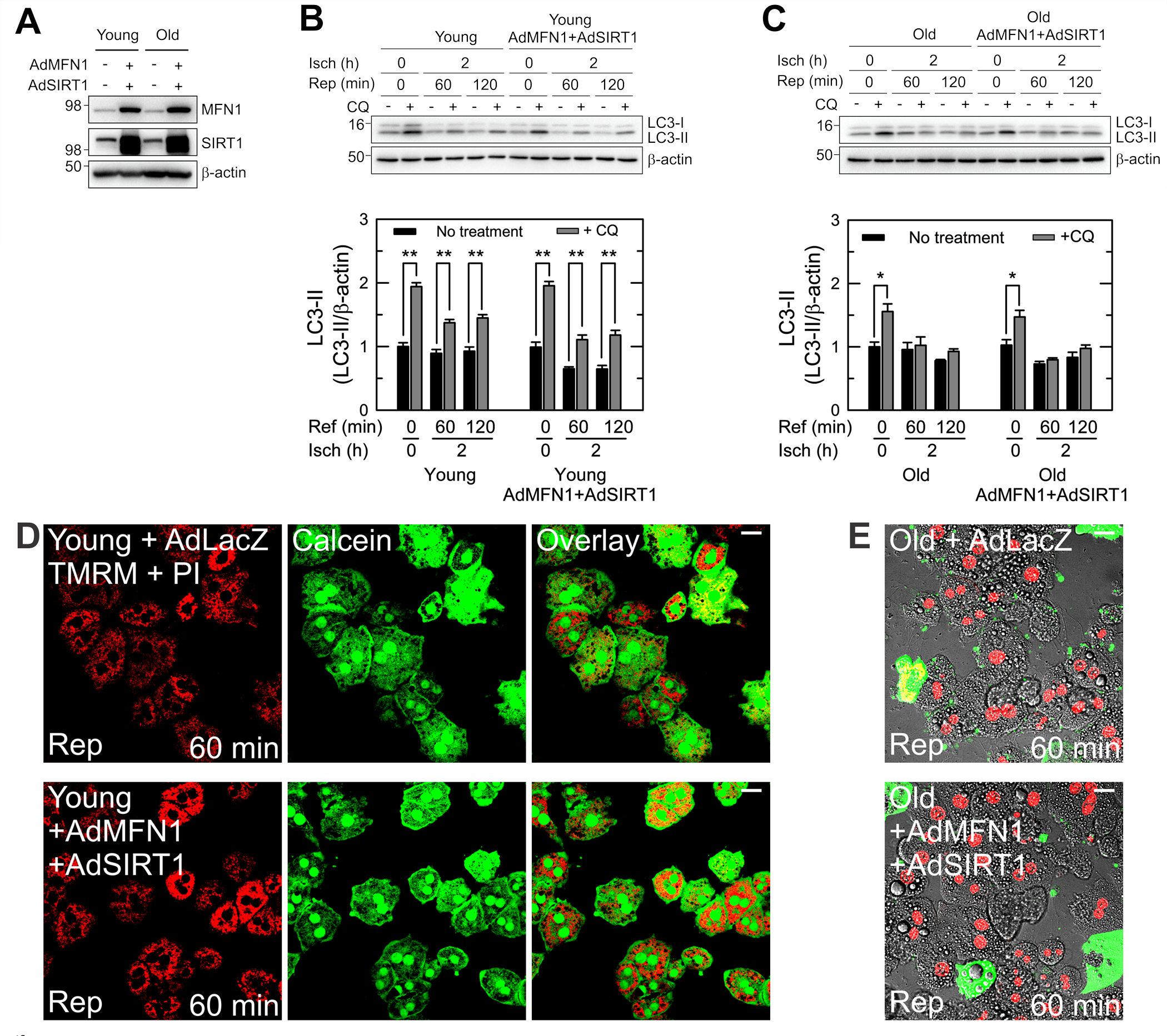

Supplement: Supplementary file 5 [file ACEL-17-na-s005.tif]
