# Supplementary material for: Loss of sirtuin 1 and mitofusin 2 contributes to enhanced ischemia/reperfusion injury in aged livers
Source: Aging Cell. 2018 May 17;17(4):e12761. doi: 10.1111/acel.12761 (PMC6052398; doi:10.1111/acel.12761)
Supplement: Supplementary file 6 [file ACEL-17-na-s006.docx]

| Gene name | Primers |
| --- | --- |
| SIRT1 | 5’- ACATCTCATGATTGGCACCG -3’  5’- GCCACAGCGTCATATCATCC -3’ |
| MFN2 | 5’- GCCTCTCTGACGTCTAGGAC -3’  5’- CAGGAGGCCATACAGTCCAA -3’ |
| GAPDH | 5’- TGGCCTCCAAGGAGTAAGAA -3’  5’- TGTGAGGGAGATGCTCAGTG -3’ |

## Table S1. Primers for RT-PCR
