# Supplementary material for: Loss of sirtuin 1 and mitofusin 2 contributes to enhanced ischemia/reperfusion injury in aged livers
Source: Aging Cell. 2018 May 17;17(4):e12761. doi: 10.1111/acel.12761 (PMC6052398; doi:10.1111/acel.12761)
Supplement: Supplementary file 7 [file ACEL-17-na-s007.docx]

**Legends to Supplementary Figures**

**Figure S1.** (A) ALT was measured after 48 h of reperfusion in young and old mice. **P < 0.01. RT-PCR was conducted to determine changes in mRNA levels of (B) SIRT1 and (C) MFN2 in young and old hepatocytes under the condition of normoxia, anoxia, and different pH. Levels of mRNA of SIRT1 and MFN2 were normalized to GADPH mRNA.

**Figure S2.** Hepatocytes were isolated from (A) young WT and (B) liver-specific SIRT1 KO mice. Confocal images of ΔΨ_m_, MPT and cell death in WT and SIRT1 KO hepatocytes were collected during normoxia (left panels) and 2 h of ischemia followed by 60 min of reperfusion (right panels). While WT cells did not undergo mitochondrial depolarization and MPT onset, KO cells showed a widespread onset of MPT and cell death (arrows) after reperfusion. Note a lack of MPT onset and cell death in KO cells under the normoxic condition. Scale bar=20 μm. (C) Immunoblotting analysis of SIRT1 and MFN2 in young WT and liver-specific SIRT1 KO hepatocytes. (D) Cell death in SIRT1 KO hepatocytes was determined after 2 h of ischemia with or without MFN2 overexpression. (E) Subcellular fractionation was conducted to separate the cytosol (C) from mitochondria-enriched membrane (M) in young and old hepatocytes.

**Figure S3.** Young hepatocytes were treated with and without AdSIRT1 and their membrane fractions (200 mg) were immunoprecipitated with the acetyl-K antibody. Immunoblotting was then conducted with putative SIRT1 targets, including MFN1, VDAC, FOXO1, FOXO3A, mitoNEET, and PGC1α.

**Figure S4.** (A) Changes in MFN1 expression were assessed after I/R in young and old hepatocytes with immunoblotting analysis. The graph represents quantification of MFN1 expression relative to the value at 0 h of ischemia in young hepatocytes. (B) Cell death in old hepatocytes was determined after 2 h of ischemia with or without MFN1 overexpression. *P < 0.05.

**Figure S5.** (A) Adenoviral overexpression of MFN1 and SIRT1 was confirmed in young and old hepatocytes with immunoblotting analysis. Autophagic flux in (B) young and (C) old hepatocytes after I/R was assessed in the presence and absence of co-overexpression of MFN1 and SIRT1. (D) Confocal analysis of MPT and cell death was conducted in reperfused young hepatocytes with calcein, TMRM, and PI. Note that co-overexpression of MFN1 and SIRT1 did not affect young cells. (E) Confocal and transmitted images of calcein, TMRM and PI were collected in old hepatocytes after 60 min of reperfusion with and without co-overexpression of MFN1 and SIRT1. While most cells were dead, as judged by PI labeling in the nucleus, co-overexpression failed to improve viability. Live cells became labeled with green fluorescence of calcein in its cytoplasm. Scale bar = 10 μm.

**Table S1.** Primer sequences of SIRT1, MFN1, and GAPDH for RT-PCR analysis.
